# Distinguishing cells using electro-acoustic spinning

**DOI:** 10.1038/s41598-023-46550-w

**Published:** 2023-11-22

**Authors:** Tayebeh Saghaei, Andreas Weber, Erik Reimhult, Peter D. J. van Oostrum

**Affiliations:** 1 Department of Bionanosciences, Institute of Biologically Inspired Materials, University of Natural Resources and Life Sciences, Muthgasse 11-II, 1190 Vienna, Austria; 2 Department of Bionanosciences, Institute of Biophysics, University of Natural Resources and Life Sciences, Muthgasse 11-II, 1190 Vienna, Austria; 3 London Centre for Nanotechnology, Faculty of Maths & Physical Sciences, University College London, Gower Street, London, UK

**Keywords:** Applied physics, Biological physics

## Abstract

Many diseases, including cancer and covid, result in altered mechanical and electric properties of the affected cells. These changes were proposed as disease markers. Current methods to characterize such changes either provide very limited information on many cells or have extremely low throughput. We introduce electro-acoustic spinning (EAS). Cells were found to spin in combined non-rotating AC electric and acoustic fields. The rotation velocity in EAS depends critically on a cell's electrical and mechanical properties. In contrast to existing methods, the rotation is uniform in the field of view and hundreds of cells can be characterized simultaneously. We demonstrate that EAS can distinguish cells with only minor differences in electric and mechanical properties, including differences in age or the number of passages.

## Introduction

Cell manipulation is a topic of great interest in many fields of biology, medicine, agriculture, and biophysics. As a fundamental technique to manipulate cells, cell rotation plays a key role in cell injection/enucleation^1, 2^, drug discovery, and cell phenotype characterization^3^. Rotational manipulation of cells is used for their characterization and discrimination based on morphology, membrane rigidity, viscoelasticity, electrical properties, and chemical composition^4–6^.

Cell mechanical and electrical properties can serve as label-free biomarkers to reveal their physiological status^7^. For instance, red blood cells (RBCs) infected by *Plasmodium falciparum*, which causes malaria in humans, suffer from reduced deformability; *P. falciparum* produces cytoadherence-related neoantigens that increase the rigidity and internal viscosity of the membrane. Cell electrical properties reflect the characteristics of membrane morphology, ion channel status, nucleus size, and cytoplasm conductivity^6, 8^.

Currently, the mechanical properties of individual cells can be probed with ~1000 cells per second using morpho-rheological phenotyping or deformation cytometry. The cell deformations driven by pinched flows are conceptually simple. Still, this method has the disadvantage that it does not allow probing the deformation of cells over a broad range of frequencies. Other techniques, such as differential dynamic microscopy (DDM), which allows studying the response to thermal fluctuations, or atomic force microscopy (AFM), which probes a single cell at a time, are slow, costly, and require sample preparations that may induce artefacts^9, 10^.

Existing cell rotation techniques use a variety of mechanisms, including mechanical force (in contact methods)^11^, magnetic^4, 12^, electric^13, 14^, optic^15, 16^, acoustic^17, 18^, and hydrodynamic fields^19, 20^. Conventional contact manipulation methods for the rotation of a cell use complex control systems and tools, while conventional non-contact manipulation methods have limitations regarding the probed volume and range of the rotated cell size^3, 13^.

Among the existing cell rotation and reorientation methods, electric field-based methods have been used mainly in cell analysis and characterization^8, 13, 21^. Electric field-based methods are convenient to operate, label-free, and combined with low-cost microfluidic platforms^3, 22^.

We first review the current understanding of the electrorotation phenomenon of cells and, after that, introduce Electro-Acoustic Spinning (EAS) as a related alternative technique. We introduce EAS as a high-throughput method to simultaneously probe the mechanical and electrical properties of many cells over a broad range of frequencies. Our approach combines electric and acoustic fields to resolve the limitations plaguing current characterization techniques, such as electrorotation.

Cells in electric fields are polarized and acquire electric dipoles, which are subject to dielectrophoresis (DEP) forces or torques in electric field gradients^14, 23^. The initial phenomenon observed due to DEP forces was the alignment of polarized objects in chains^23, 24^. In electrorotation (ER), the development of which started with Holzapfel et al.^24^, cells are characterized by observing the rate at which they rotate at the central axis of a uniform, rotating AC electric field. Here, the phase difference between the induced dipole moment of the cell and the rotating electric field generates a torque. The cells rotate in the plane in which the electric field rotates. Novel ER configurations have been developed that enable 3D rotation on a chip with a high degree of control^6, 13^. The electrical parameters of individual cells, such as membrane capacitance and cytoplasmic conductivity, can be measured by analysing the rotation spectrum, which maps the rotation speed of the cells as a function of the speed of rotation of the field^14, 24^. Still, it comes at the cost of a severely limited throughput^13^.

Also cell rotation in non-rotating electric fields has been reported and investigated, but the underlying mechanism is not fully understood. Conventional DEP with two electrodes inducing *non-uniform* AC electric fields was reported to induce rotation in different cells, e.g., yeast^23, 25^, rat adipose stem cells^26^, Melan-A cells^27^, lymphocytes, white blood cells^28^, melanin pigmented cells^29^, promyelocytic leukemia cells taking up nanoparticles^30^, Jurkat, HEK, and PC3 human cell lines^31^.

DEP forces and torques are proportional to the cube of the cell radius. Therefore, ER and other DEP methods have only been applied to cells bigger than ~10 µm. Another challenge with DEP methods is that the cell spinning speed depends on the cell’s position in the electric field. This makes it very complex to measure the electrical parameters of cells accurately^13, 27^.

There are very few reports of cell rotation in *uniform, non-rotating* electric fields^23, 25, 32, 33^. Teixeira-Pinto et al. were the first in 1960 to report the spinning of microorganisms in a non-rotating high-frequency field (100 kHz-100 MHz)^33^. The aggregated cells started rapidly rotating once they came close to the wall. Zimmerman et al.^24, 34–37^ in the 1980s observed electrorotation between two parallel electrodes of biological cells such as mesophyll protoplast cells of *Avena sativa* (at 20–40 kHz), erythrocytes, ghost cells (at 80–100 kHz), and yeast cells (at 140–180 kHz). They showed that reproducible rotation of biological cells was achievable during the dielectrophoretic formation of cell chains. At least two cells had to be in close proximity for rotation to occur^34^.

These observations led Holzapfel et al.^24^ in 1982 to suggest that the rotation was due to an interaction between the dipoles induced in each cell. They hypothesized a rotating component to the local field that depends on the relative positions of the cells by implicitly adding the phase-shifted electric field associated with the induced dipole near each cell to the applied field^36^.

Many researchers in the electrorotation field questioned whether or not single-cell spinning in a uniform electric field is even possible. Turcu presented an analytical model in 1987 showing a spherical rotor rotating in a uniform AC electric field^38^. He suggested that Brownian motion played the role of a disrupting factor to begin a rotation that would subsequently be sustained by the torque the external field exerts on the reoriented induced dipole. In the late 1990s, Krause et al.^39^ observed the rotation of polystyrene (PS) drops dispersed in a polydimethylsiloxane (PDMS) medium at 0.1 Hz and 1 MHz AC fields. They found that Turcu's theory did not describe their experimental results and suggested that the liquid droplets' fast rotational speed might be related to their deformability, a factor that is not considered in Turcu's model.

As the exposé above reveals, it is unclear how we can explain the many similar observations of the rotation of cells exposed to differently generated electric fields. Specifically, due to many experimental challenges and difficulties reproducing earlier experimental results, the spinning of single objects in uniform AC fields remains a controversial and poorly understood topic. Experimental observations for spinning objects in uniform AC electric fields are limited. In previous reports, the researchers presented the spinning as an interesting side observation in experiments conducted with other aims^25, 32, 33^. The only quantitative study was reported by Zimmerman et al. In their study, Zimmerman et al.^34^ rejected the possibility of single objects spinning inside a uniform part of the AC electric field. The many poorly described and sometimes contradictory observations^23, 24, 32, 33, 39, 40^ have given rise to equally conflicting theories about their origins.

We hypothesize that acoustic fields created by the periodic attractions between the electrodes or shape oscillation of nearby cells interplay with the electric field-induced shape oscillations of deformable cells to cause the rotation observed in some experimental setups but not others. Because of the subtle interplay between AC field-induced shape oscillations and the local ultrasound field, we call this phenomenon electro-acoustic spinning (EAS).

**Figure 1:**
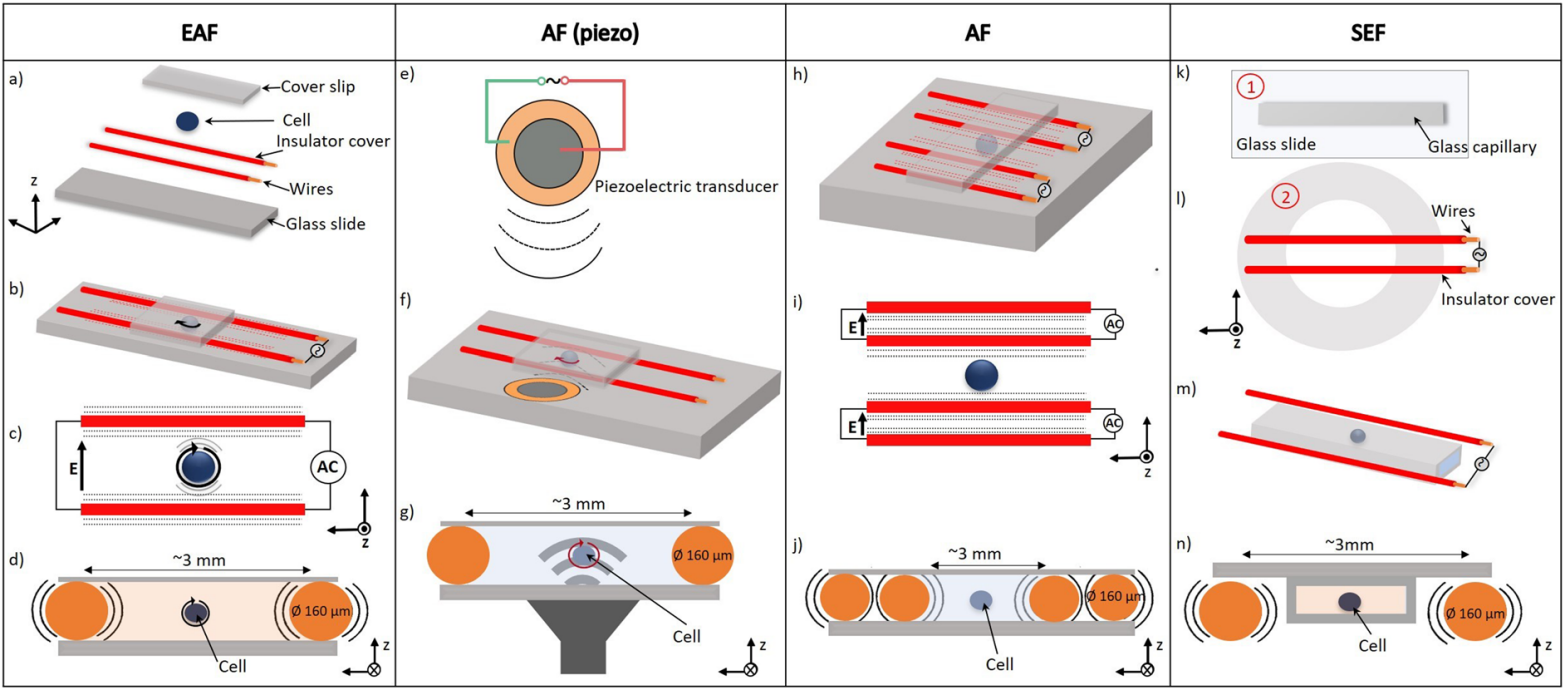
Design of the cell for the application of electro-acoustic fields (EAF), acoustic fields using a piezoelectric transducer AF(piezo) or induced by wire electrodes with a canceled electric field (AF), or silent electric fields (SEF). (**a**) Microfluidic channel components. (**b**) Electrodes in mechanical contact with the sample container. (**c**) Schematic of rotation in an EAF. (**d**) Cross-section of the capillary, the water has been coloured orange to highlight the presence of the electric field (EF). (**e**, **f**) Acoustic field applied separately using a piezoelectric transducer glued to the microscope slide next to the capillary. (**g**) Cross-section of the capillary with applied AF. (**h**, **i**) An acoustic field is applied by two pairs of vibrating electrodes with opposite polarity while the EF is cancelled in the middle of the capillary. (**j**) Cross-section of the capillary. (**k**,**l**,**m**,**n**) Design of the cell for the application of silent electric fields. (**k**, **l**) Microfluidic channel designed to apply SEF. Part 1: a capillary glued on a glass microscope slide. Part 2: a polystyrene Petri dish with a hole in the middle, two electrodes used to apply the electric field spanned over the hole, and two soft silicone supports to mount part 1. (**m**) Parts 1 and 2 mounted together, yielding electrodes not in mechanical contact with the container. (**n**) Cross-section of the capillary, the water has been coloured orange to highlight the presence of the EF.

Our hypothesis is that electro-acoustic spinning occurs if and only if there is the concurrent presence of a 1) polarizable and deformable dispersed object exposed to a 2) sufficiently strong AC electric field in combination with 3) a sufficiently strong acoustic field of the same frequency. To test this hypothesis, we used deformable oleic acid drops and hard particles in specially designed measurement chambers with or without mechanical contact between the electrodes and the sample container (Fig. 1 and S1) that allow applying either strong AC fields, strong acoustic fields or combined fields. The periodic attractions and repulsions between electrodes create a homogeneous ultra-sound field inside the capillary in the case of mechanical contact between electrodes and container (Fig. 1a–d). The ultra-sound field superimposes on the electric field and combines into an electro-acoustic field (EAF) in the sample. To test if no spinning occurs in the absence of an acoustic field, we create a 'silent' AC electric field (SEF), without the associated ultrasound, which can be created by placing the sample in a capillary between the electrodes without mechanical contact (Fig. 1k–n). To test if no spinning occurs in the absence of an electric field, purely acoustic fields (AF) are made by actuating the same sample environment using either a piezoelectric actuator (Fig. 1e–g) or using another pair of electrodes with opposite polarity to cancel the electric field in the middle of the capillary (Fig. 1h–j).

We elucidate the mechanism behind the rotation of soft objects like cells in unidirectional AC electric fields by first studying the rotation of oleic acid droplets and solid particles in an EAF or a SEF. To demonstrate the applicability of electro-acoustic spinning, we then investigate the rotation of MCF-7 and HeLa cells as a function of applied field frequency for different passage numbers and cultivation times, as fixed cells, or with depolymerized actin filaments.

## Results

**Figure 2:**
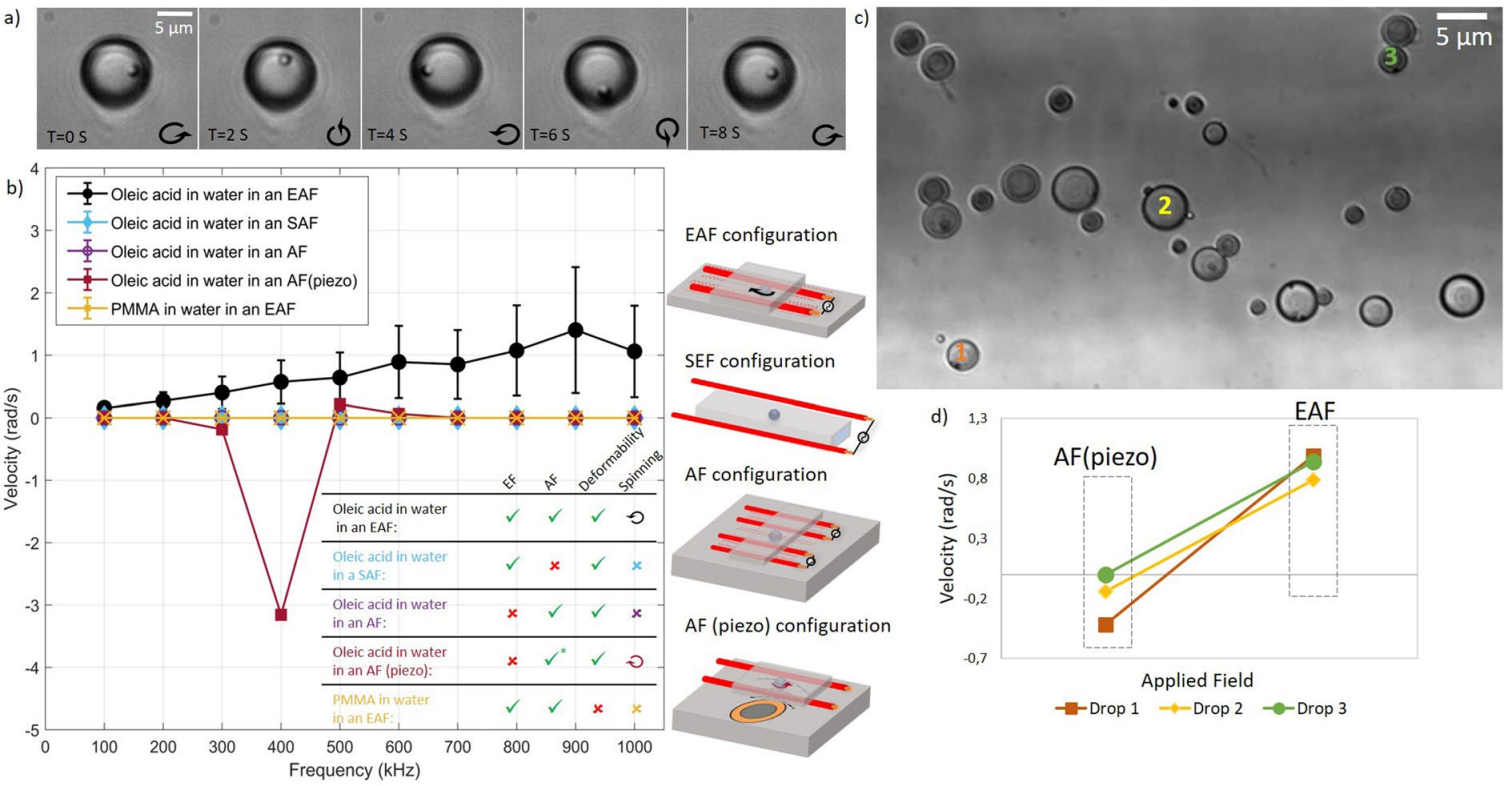
Experimental results for oil droplets subject to EAF, SEF, AF, or AF(piezo). (**a**) Rotation of an oleic acid drop in water in a 50 V/mm electro-acoustic field at 200kHz. (**b**) Rotation speed versus frequency of oleic acid droplets in EAF (black), SEF (blue), AF (purple), and AF piezo (red), and solid, rigid PMMA spheres or silica rods in EAS (yellow), demonstrating the need for the combination of the electric field with an acoustic field to induce rotation of deformable objects. (**d**) The rotation velocities of the three oleic acid droplets suspended in water enumerated in (**c**) subjected to AF (piezo) and EAF at 400 kHz, respectively. The same frequency was used for the AF(piezo) and EAF, however, the type of field, directions, spatial distribution and intensities were different.

Figure 2 shows the rotation velocity of oleic acid drops suspended in water versus the frequency in an electro-acoustic field (EAF), a silent electric field (SEF), and an acoustic field (AF). Additionally, it shows the rotation velocity of rigid poly(methyl methacrylate) microparticles (PMMA-AR145, Microparticles GmbH) in an EAF. Figure 2a shows the rotation of an oil droplet containing PS microparticles in an EAF (Movie S1), making it possible to quantify the rotation speed as a function of applied electric field frequency, as shown in Fig. 2b. In the SEF (blue line), only strings of drops formed while none of these drops rotated (see Figure S3 and Movie S2). In the AF generated by the piezoelectric transducer (red line), fast rotation was observed at a resonance frequency of 400 kHz for the oil droplets in our 2 mm wide capillary (Movie S3). However, we did not observe the droplets spinning at any frequency in the middle of the capillary when we produced the acoustic field with two pairs of wires and the electric field was canceled by a second set of parallel electrodes (Figure S5). Solid, non-deformable objects, here represented by poly(methyl methacrylate) microparticles (PMMA-COOH-AR145, Microparticles GmbH) and silica microrods (Figure S4), did not spin at any frequency in the EAF (yellow line). Importantly, oil droplets spinning over a broad range of frequencies were observed only in EAF (black line).

In Fig. 2d, we plot the speed of rotation of three drops labelled in image 2c under different field conditions to compare the influence of an EAF and a AF (piezo) using a piezoelectric transducer. The spinning of the deformable oil droplets in the EAF was distinctively different from the resonant rotation^41^ observed in an AF(piezo). The individual droplet rotation velocities at resonance in the AF(piezo) differ for the three drops. The positional velocity dependence for the similarly sized droplets 1 and 3 should be particularly noted. Droplets 1 and 3 spin at nearly the same velocity across the frequency spectrum in the EAF. Size affects the rotation speed in an EAF as droplet 2, which is positioned halfway between droplets 1 and 3, rotates slower due to its larger size. Nevertheless, our observations indicate that the influence of size is less pronounced for larger objects, Figure S6. The qualitatively similar but size-dependent spinning of droplets was observed throughout the sample volume, as exemplified in Movie S1 in the supporting information.

The rotation spectrum, i.e., rotation speed and direction as a function of EAF frequency, depends on the properties of the microfluid channel and the suspended sample. For some objects, we observed the direction of rotation change within the accessible frequency range. Additionally, we noted that changing microfluid channel features, such as electrode geometry or environment (e.g., water or air), that affect the phase difference between the acoustic and electric fields, could lead to a reversal of the rotation direction for the same object in the same frequency range. Similar objects rotate similarly, independent of their location in the microscope's field of view. Importantly, these results are reproducible under the same experimental conditions.

These observations firmly establish that for EAS, the combined effects of an AC electric field with an ultrasound field of the same frequency are required to observe rotation and that this only occurs for a polarizable and deformable object in suspension (cf. Figure S4), as summarized in the table in Fig. 2b. The same-frequency ultrasound field is conveniently induced by the mechanical contact between the sample cell and the electrodes. Note that our experimental design with the electrodes positioned outside the channel prohibits the creation of electroosmotic or electrothermal flows that could induce rotations locally.

**Figure 3:**
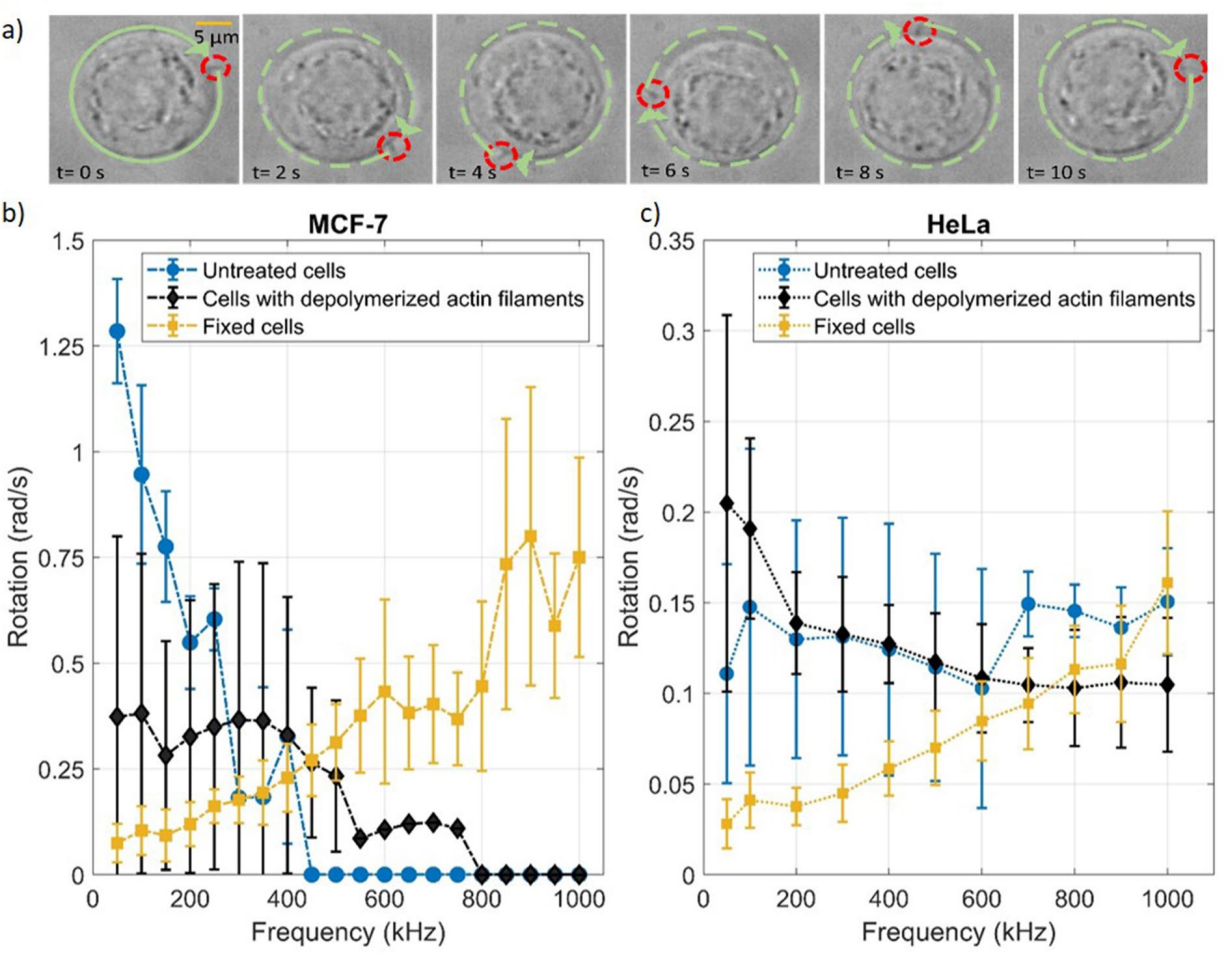
EAS of cells with different mechanical properties. (**a**) An MCF-7 cell in 25 V/mm at 100 kHz rotates at 0.63 rad/s clockwise (SI); Rotation speed of (**b**) MCF-7 cells and (**c**) HeLa cells suspension in 25 V/mm vs. frequency for normal and fixed cells, and cells with depolymerized actin filaments. Data for each point is collected from 10 to 20 cells from at least 3 repeated experiments. The plots were generated using the same number of data points for consistency. The cells were cultured for 24h and dispersed in the measurement medium.

We demonstrate that electro-acoustic spinning allows us to reproducibly distinguish suspended objects in a large volume by applying EAS to various kinds of cells. Figure 3a exemplifies the rotation of MCF-7 cells in an electro-acoustic field applied with the electrode configuration shown in Fig. 1b (Movie S4).

The rotation velocity spectra of MCF-7 and HeLa cells, respectively, exposed to EAF frequencies between 50 and 1,000 kHz at constant field strength, are shown in Fig. 3b and c, respectively. The cells have been treated to have different mechanical properties, i.e., either left untreated, with depolymerized actin filaments, or with crosslinked proteins (fixed). The cell rotation velocity changes with the frequency of the EAF in all samples. Each cell type and cell treatment showed a unique rotation velocity spectrum. The cells are nearly uniform in size and sufficiently large, < 15 µm, such that the impact of size on their rotational behavior can be considered negligible. Untreated MCF-7 cells and cells with depolymerized actin filaments rotate faster when exposed to electric fields of lower frequency, while fixed MCF-7 cells rotate faster at higher frequencies. Although both untreated and MCF-7 cells with depolymerized actin filaments exhibit decreasing rotation with increasing EAF frequency, the MCF-7 with depolymerized actin filaments rotate slower but over a broader frequency range than the untreated cells.

The rotational velocity of untreated HeLa cells is more or less constant in the 50 and 1000 kHz frequency range (Fig. 3c). For fixed HeLa cells, the velocity increases with increasing frequency. Cells with depolymerized actin filaments decrease their rotation velocity slightly with increasing frequency. In general, HeLa cells rotate slower than MCF-7 cells in the probed EAF frequency range. Clearly, the rotation velocity spectrum of cells is influenced by the mechanical properties, as expected if the mechanical deformation of the cells in the acoustic fields is key to the rotation. The general trend is that fixed cells that are presumably stiffer show increasing rotation velocity with increasing frequency in the kHz to MHz range, while cells with lower stiffness show the opposite trend and can even stop rotating at high frequencies.

Cells change their physical properties, including their mechanical and electrical properties, over their life cycle^42, 43^. AFM measurements show that the number of passages affects the mechanical properties of cells. Usually, 2–8 passages are used to perform reproducible experiments^44–46^. Given the sensitivity of EAS to a cell's mechanical properties, we expect to distinguish cells in various stages of development with our technique.

**Figure 4:**
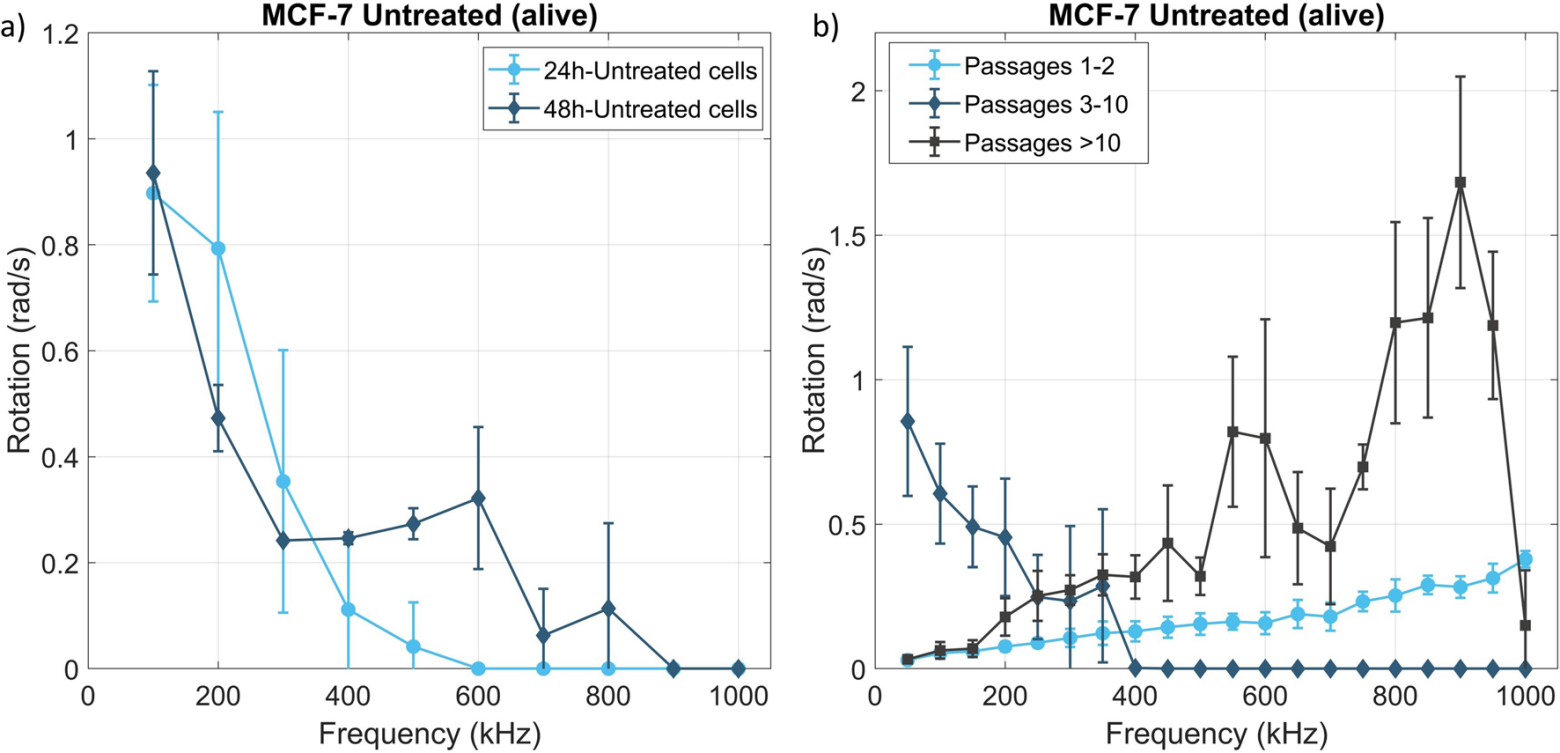
The effect of age and passage on EAS. Rotation speed of MCF-7 cells in 25 V/mm vs. frequency (**a**) for 2 different growing times, and (**b**) for different numbers of passages grouped into passage intervals with distinguishable EAS behaviour. Data for each point is collected from 10 to 20 cells from at least 2 repetitions. The plots were generated using the same number of data points for consistency.

Figure 4a shows the rotation spectra of untreated MCF-7 after different cultivation times. Longer (48 h) growing time increases the EAF range over which rotation is observed. Figure 4b shows the rotation spectra of MCF-7 cells as a function of the number of times they have been passaged. Cells passaged 1 or 2 times have low rotation velocities and rotate slightly faster with increasing EAF frequency. In contrast, cells passaged 3–10 times show high rotation velocities at low EAF frequencies within the probed range, dropping rapidly to zero above 400 kHz. After passage 10, the behaviour again changes significantly, and the rotation speed dramatically increases with the applied EAF frequency.

In summary, our results show that the rotation velocity spectrum of cells in an EAF can be used to distinguish their development and age. Previously published AFM data^44^ demonstrate that these cells change their stiffness with passage generation. Likewise, we observe that MCF-7 cell passaged once or twice have qualitatively and quantitatively different EAS spectra to cells passaged more times. The observed differences are reminiscent to those observed between untreated and fixed cells, i.e., cells passaged many times are seemingly mechanically stiffer. This implies that the change in EAS spectra for the MCF-7 cells with respect to passage number is related to changes in their mechanical properties.

## Discussion and conclusions

After more than half a century of somewhat conflicting research results on the topic, our work (re)introduces the rotation of deformable objects in homogeneous AC electric fields as an electro-acoustic phenomenon. We propose a mechanism (the combined effect of AC electric field-induced shape oscillations with an ultra-sound field of the same frequency generated by the motion of the cyclically attracting electrodes) to explain electro-acoustic spinning, i.e., the phenomenon of cell spinning in electro-acoustic fields. Negative control experiments strongly support our hypothesis. Firstly, non-deformable solid objects do not rotate in an EAF, demonstrating that mechanical deformation is necessary. Secondly, we do not observe any rotation of objects in experiments performed in silent electric fields generated without mechanical contact between the electrodes and the capillary (Fig. 1k–n and 3S). We also do not observe it in acoustic fields produced by the vibration of electrodes in mechanical contact with the sample container, for which the electric field was canceled by an additional pair of opposing wire electrodes (Fig. 1h–j and S5). However, we observe the rotation of cells and other deformable objects (Figure S4) in a homogeneous AC electric field applied to the capillary with the electrodes in mechanical contact with the capillary (Fig. 2 and 1a–d). The electrodes are only in contact with the outside of the suspension container and not in direct contact with the suspension. The only difference between the EAF and SEF configurations is that with mechanical contact, the electric field causes mechanical movement at the frequency of the electric field, giving rise to an acoustic field within the capillary. Hence, the superposition of an acoustic and an electric field of the same frequency is responsible for the observed rotations. Thirdly, EAS is qualitatively and quantitatively distinct from the rotation of deformable objects in a purely acoustic field generated by a piezoelectric actuator. Rotation in an AF(piezo) is a resonant phenomenon (Movie S3), leading to different rotational speeds of the same object at different locations in the sample volume, in contrast to the reproducible spinning at any location observed for EAS.

We can outline the essential parameters for EAS, although our experiments do not allow for the formulation of a quantitative description of EAS based on the physical properties of the spinning objects. In our setup, objects are deformed by the acoustic field generated by the electrode movements. As the sound waves pass through the suspension, they exert acoustic pressure on the suspended objects^47^. This results in the deformation and motion of the object. The magnitude depends on the density difference between the object and the solvent, the size, shape and compressibility of the object, and the frequency of the sound wave^48, 49^.

A deformable object placed in a uniform electric field adopts a prolate or oblate spheroidal equilibrium shape around which the shape oscillates at the frequency of the applied field. These shape oscillations induce flows around the object that are axisymmetrically aligned with the applied field. The objects deform and oscillate due to the AC electric field as they are elongated or compressed depending on the sign of the object's polarizability and due to flows associated with electro-osmosis near the object’s charged surface^48, 50^.

There is a phase difference between the electric and acoustic fields, which varies only on the length scale of the shortest involved waves, in this case, the millimetric ultrasound waves. Hence, this configuration affects suspended particles in a large volume quantitatively similarly. The phase difference between the deforming fields translates into a net torque on the suspended objects during each period of the EAF, which results in a steady rotational movement. This may explain many of the described yet poorly explained and sometimes contradictory observations in the literature^23, 24, 32, 33, 39, 40^.

Based on our interpretation of the effect of the acoustic field, the mechanical properties of the sample cell and the individual biological cells critically influence the spinning velocity. The radiation pressure depends on the density and viscosity of the medium and the object. For the electric field-induced deformation, the electrical properties such as conductivity, polarizability, and charge of the cell determine the strength of the deforming force. Together with the viscosity and elasticity, they determine the equilibrium shape and shape oscillations in an AC electric field. The practical inter-dependency of many of these parameters when constructing an experiment makes it difficult to elucidate their quantitative influence individually, but it should be possible to formulate a comprehensive theory.

Our work introduces electro-acoustic spinning as a simple and sensitive method to study suspended colloids with particularly useful applications for analyzing biological colloids such as cells. The EAS spectra of cells depend sensitively on the individual cells’ electrical and mechanical properties, allowing discriminating cells that differ only by age, passage, and treatments in a reproducible manner.

The spinning velocity, including the direction of rotation, could be controlled by adjusting the applied electric field strength and frequency in the same sample cell. Furthermore, the spinning velocity quantitatively depended on experimental parameters, such as the ionic strength, providing additional tunability for EAS as a method for discriminating bio-samples. A main advantage of electro-acoustic spinning over previous demonstrations of electrorotation for cell analysis is that it makes use of homogeneous electric fields. With our explanation, which postulates a torque resulting from the phase difference between the acoustic and the electric fields acting on a suspended object, the volume within which cells are exposed to the same forces is determined by the wavelength of the ultrasound at the applied frequency.

The acoustic wavelength in water at kHz frequencies is larger than the field of view of a typical light microscope. Thus, multiple rotating cells distributed throughout a large sample volume can be observed simultaneously. The quantitatively similar spinning of the same type of objects in a large volume in an EAF distinguishes EAS from previous works. It allows us to monitor and collect data on multiple objects simultaneously, improving throughput. The EAS is observed in single objects positioned both far from the electrodes and each other, as well as in neighboring objects forming strings due to the electric field (Movie S1 and S4). The operation of EAS requires less skill, equipment, and patience to position and keep cells on the axis of rotation of the electric field and greatly enhances the throughput of EAS compared to conventional electrorotation.

Our current version of EAS also has limitations. EAS can only be used to probe objects deformed by the applied acoustic and electric fields in suspensions with low conductivity. For the purpose of measuring the physical parameters of individual cells from EAS spectra quantitative models describing the rotational spectrum of every relevant type of object in an EAF as a function of its properties are needed, this is beyond the scope of this work and subject of further study.

With these caveats and the need to further develop EAS for applications, we confidently infer that EAS shows all the characteristics to be a high-resolution, label-free, high-throughput technique to characterize cells and other polarizable colloids based on their electro-mechanical properties.

## Materials and methods

### Measurement cell design and electrorotation measurements:

The microfluidic channel for the experiments with the electro-acoustic fields (EAFs) is shown in (Fig. 1a–d). It was built by gluing two pieces of enamelled copper wire (1,567,045 TRU COMPONENTS-150–160-μm) on a microscope slide (Carl Roth- NK72.1) using optical adhesive (NOA81 Norland) as walls of the capillary and closed on top with a coverslip (Carl Roth- H873) using the same glue. The electrodes are not in direct electrical contact with the solution medium to avoid unwanted electrothermal flows caused by Joule heating. For the control experiments with the silent electric fields (SEFs), the sample is contained in a capillary (Vitrocom Inc. 2 mm width) mounted between two wires with minimal mechanical contact to the electrodes to not expose the sample to the acoustic vibrations caused by the electrodes (Fig. 1k–n). The parallel wires produce a linearly polarized electric field inside the channels (Fig. 1d, n). To apply a purely acoustic field, a piezoelectric transducer (KPT-G1420A-K8437) is glued next to the capillary (Fig. 1e–g). The capillaries are mounted on an upright microscope (Nikon, Eclipse TS100) equipped with a 40x (Nikon E-plan NA: 0.65) objective to capture live bright-field images during the experiment. The same capillary size and geometry with 3 mm width and 2 cm length were used for all experiments with EAFs (Figure S1). The sample dispersions are sucked into the sample cell using capillary forces upon pipetting them to the end of the capillary. The sinusoidal AC signal is generated by a wave function generator (TTI TG1010A-10 MHz DDC) that is connected to an amplifier (KROHN-HITE 7602 M. 1 MHz-34W-800Vpp). This sets the higher end of the frequency range we can probe while increasing the frequency would further shorten the wavelength of the ultra-sound to close to the extent of a microscope’s field of view. The speed of rotation was determined by tracking objects in each frame.

### Oil suspension preparation

The suspensions were prepared by adding oleic acid (90%, 364,525 Sigma-Aldrich) to DI water at room temperature. The mixtures were shaken for a minute to separate the oil into polydisperse droplets. 1 µm polystyrene particles were added to the oil in advance to enable tracking the rotation of the droplets. The results for each data point were collected from at least 3 repeated experiments and averaged over 10–20 drops for similar sizes of the drops (12–13 µm).

### Cell preparation

MCF-7 epithelial breast cancer cells were a gift from Maria dM Vivanco (CIC bioGUNE). They were grown in DMEM supplemented with 10% fetal bovine serum (FBS) and 1% PenStrep. HeLa CCL2 (HeLa ATCC CCL-2) cells were cultured in MEM supplemented with 10% FBS and 1% PenStrep. All cell culture media and chemicals used were supplied by Thermo Fisher Scientific. Both cell lines were passaged twice per week at a maximum confluence of 80%. The cells were trypsinized and counted before each measurement. They were then centrifuged, the supernatant removed, and washed once with PBS. The cells were resuspended at a concentration of 10^5^ cells/mL in a medium consisting of 0.05% BSA, 200 mmol/L mannitol, 50 µmol/L CaCl_2_, 100 µmol/L MgCl_2_, and 500 µmol/L Hepes^51^ to enable measurements by limiting the screening of the electric fields in the measurement chamber by ions. Cell viability in the measurement medium was confirmed for at least 24 h. Cells were treated with 5 µmol/L cytochalasin D for 30 min at 37 °C in the culture medium to depolymerize the actin filaments before resuspending them in the measurement medium. Fixed cells were prepared by treatment with 4% paraformaldehyde at 37 °C for 10 min, followed by centrifugation and resuspension in the measurement medium. These treatments of the cells affect their mechanical properties. The cells used in all experiments were passaged 3–10 times, except when reported otherwise. Data for each data point was collected from at least three biological replicates, and multiple experiments were conducted to gather velocity data from 10–20 cells per data point. The obtained data were then averaged over these cells for each data point.

### Supplementary Information

Supplementary Information 1.

Supplementary Video 1.

Supplementary Video 2.

Supplementary Video 3.

Supplementary Video 4.

## Data Availability

The datasets used and/or analysed during the current study available from the corresponding author on reasonable request.
