# Supplementary material for: Distinguishing cells using electro-acoustic spinning
Source: Sci Rep. 2023 Nov 22;13:20466. doi: 10.1038/s41598-023-46550-w (PMC10665424; doi:10.1038/s41598-023-46550-w)
Supplement: Supplementary file 1 — Supplementary Information 1. [file 41598_2023_46550_MOESM1_ESM.docx]

Distinguishing cells using Electroacoustic Spinning

# Supplementary Information

Tayebeh Saghaei,*^a^ Andreas Weber,^b^ Erik Reimhult ^a^ and Peter D. J. van Oostrum^a^


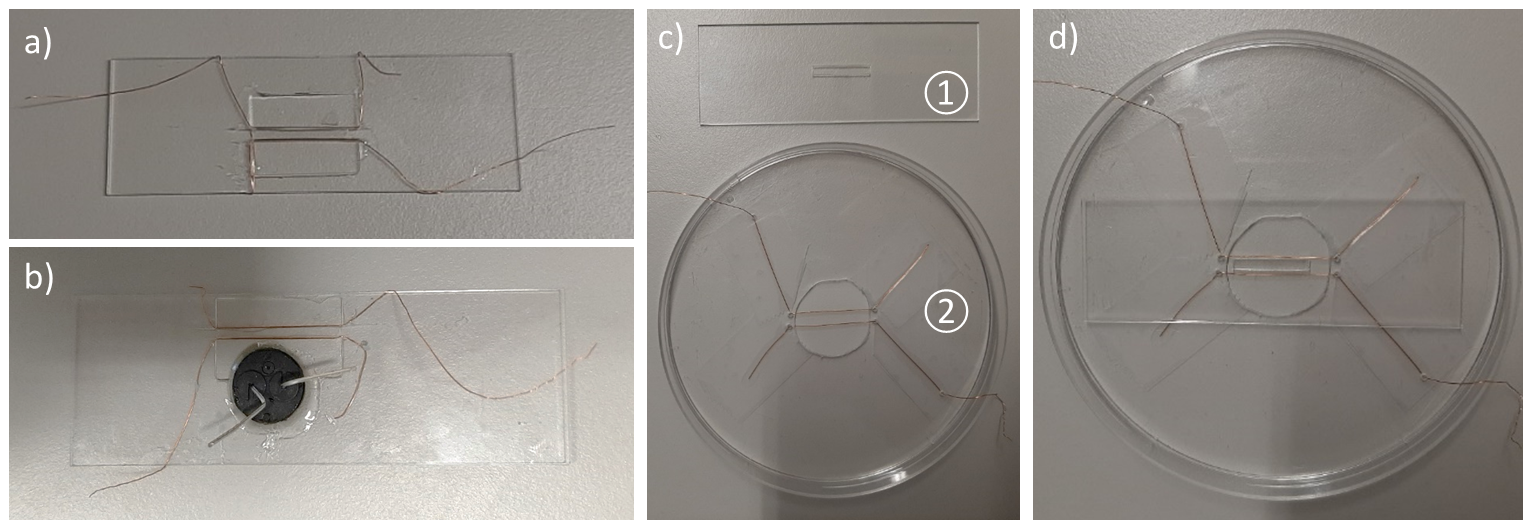


Figure S1: Pictures of the used sample cells: a) Image of the sample cell for electroacoustic field application (EAF). b) Sample cell for the application of an acoustic field applied separately using a piezo speaker glued to the microscopy slide next to the capillary. c) Sample cell for the application of a silent electric field (SEF) Part 1: a capillary glued on a glass microscopy slide. Part 2: a polystyrene Petri dish with a hole in the middle, two electrodes used to apply the electric field span the hole, and two soft silicone supports mount part 1. d) Parts 1 and 2 together.

**AFM method:** MCF-7 cells (either control, after 30 min treatment with 5 µM Cytochalasin D, or fixed for 10 min with 4% PFA) were centrifuged, washed once with PBS and once with the measurement medium. Thereafter, the cells were diluted to 1x10^5^ cells/mL in the medium. Round glass slides (Menzel Gläser, 24 mm diameter, strength of 1) were cleaned with EtOH, dried with N_2_, plasma cleaned with oxygen plasma, and functionalized with 0.01% PLL for 1 hour. Prior to measurements, they were washed with PBS. For measurements, a JPK Nanowizard III (Bruker, Germany) with a CellHesion extension placed on an inverted optical microscope (Zeiss AxioObserver Z1, Zeiss, Germany) was used. A liquid, temperature-controlled sample stage was employed. Measurements were performed at 37 °C in the measurement medium. Tipless NP-O cantilevers (cantilever B, nominal stiffness of 0.12 N/m, nominal resonance frequency in air of 23 kHz) functionalized with silica particles of 10 µm diameter were cleaned for 30 min in a UV/Ozone chamber and calibrated before measurements using the thermal noise method^1^. For measurements, 500 µL of the cell suspension was added to the sample holder and left to sediment for 10 min, then the AFM head was added, the surface was approached, and the system was equilibrated for 30 min. The microscope was then used to define measurement positions on top of each cell. The curves were measured using an approach and retract velocity of 5 µm/s, a curve length of 10 µm, a sampling rate of 2048 Hz, and a maximum force of 2 nN (approx. 1 µm indentation). Each cell was measured at least 2 times, and at least 10 cells were measured per condition. The whole experiment was repeated twice. For data evaluation, curves were extracted using the JPKSPM software (JPK, Bruker, Germany) and processed with the R package *afmToolkit^2^*. Contact and detachment points were calculated, baselines were corrected, and the cantilever deformation was determined. Assuming cells behave as isotropic linear elastic bodies for small deformations, the Young’s Modulus of the cells was determined using 500 nm of the force-indentation curve as

$$F=\frac{4}{3}\sqrt{R_{c}}\frac{E_{app}}{1-\nu^{2}}\delta^{\frac{3}{2}},$$

where $F$ is the applied force, $R_{c}$ the radius of the indenter (tip radius), $E_{app}$ the apparent Young’s Modulus, $\nu$ the Poisson’s ratio (set to 0.5), and $\delta$ the deformation of the sample.


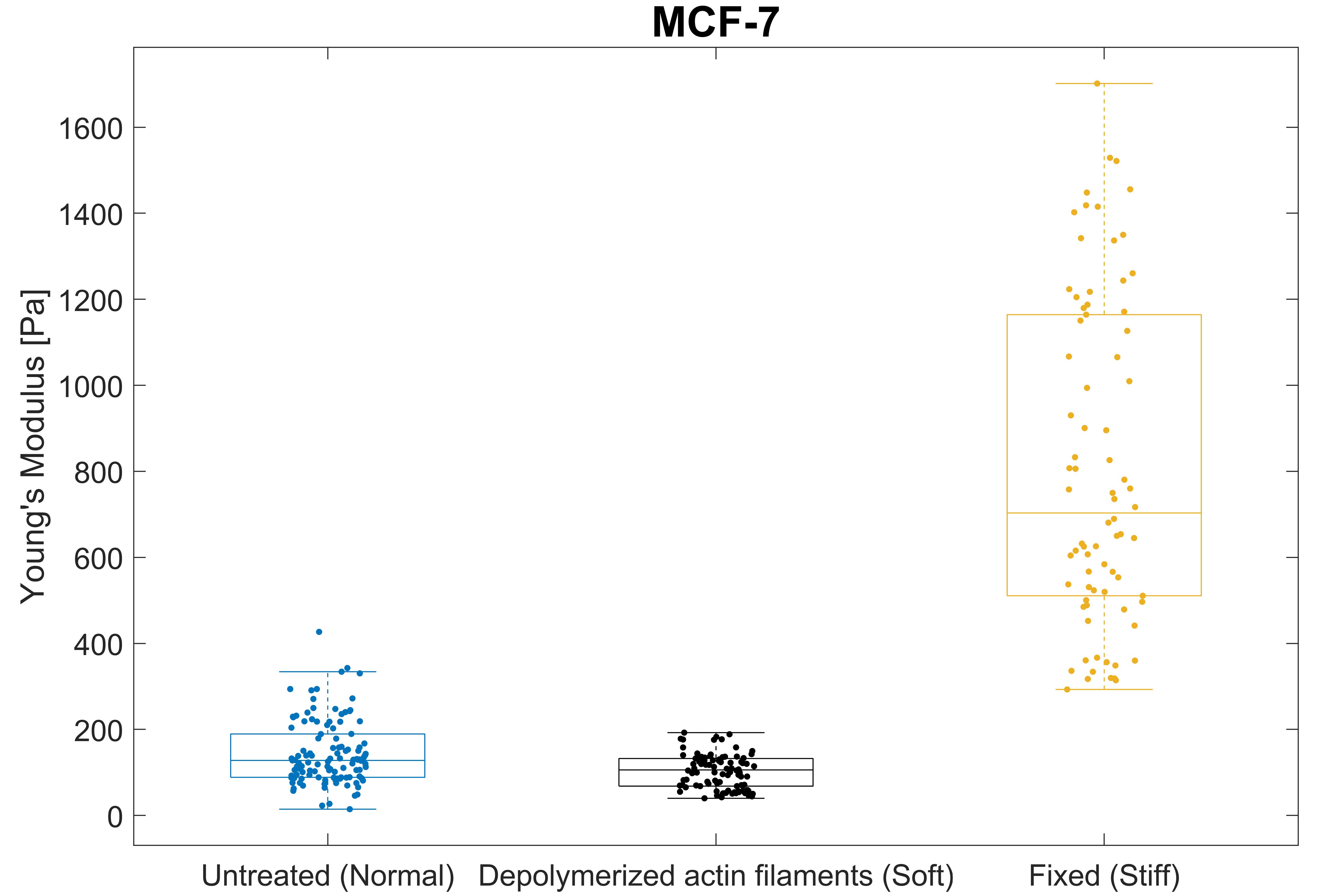


Figure. S2: Young’s modulus of untreated MCF-7 cells, MCF-7 cells with depolymerized actin filaments and, fixed MCF-7 cells are measured using AFM with 5 µm/s speed.

| Sample | Zeta potential (mV)  MCF-7 | Zeta potential (mV)  HeLa |
| --- | --- | --- |
| Untreated cells (alive) | -21,2 | -23,2667 |
| Cells with depolymerized actin Filaments (soft cell) | -22,9 | -23,2 |
| Fixed cell (stiff) | -18,56 | -24,3333 |

Table S1. Zeta Potential of cells in the medium.


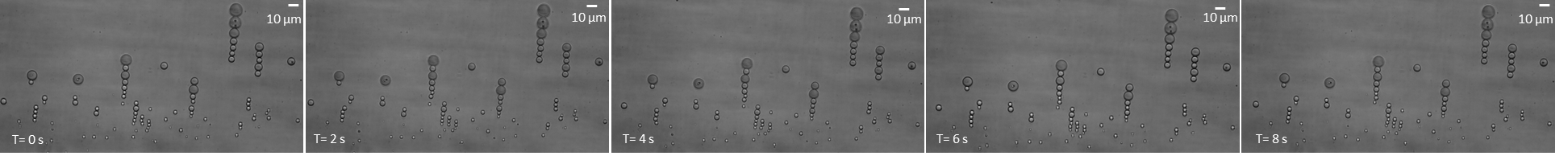


Figure. S3: String formation of oleic acid drops in water, without rotation, in a silent electric field at 50V/mm and 400kHz (Movie S2).

**Polymer coating on silica rod:** 0,1 mg of silica rod particles was dispersed in 10 mL of a 10% 3-aminopropyldimethylethoxysilane (APDMES) (Mw = 161g/mol, purity ≥ 97%, AB110423) was purchased from abcr (Karlsruhe, Germany), mixed in methanol and stirred overnight. The aminated particles were washed with methanol and then water. Next, the particles were dispersed in hexamethylene 1-6- diaminocarboxclsolfunat solution in water (1 mmol/L) and sonicated for 30 min. After washing the particles with water 3 times, they were dispersed in a poly(ethyleneimine) (9002-98-6, Mw ~750,000 by LS, 50 wt. % in H2O, Sigma-Aldrich) solution in water (1 µmol/L) and stirred for 2h. Particles were washed with MilliQ water 3 times and dispersed in MilliQ water.


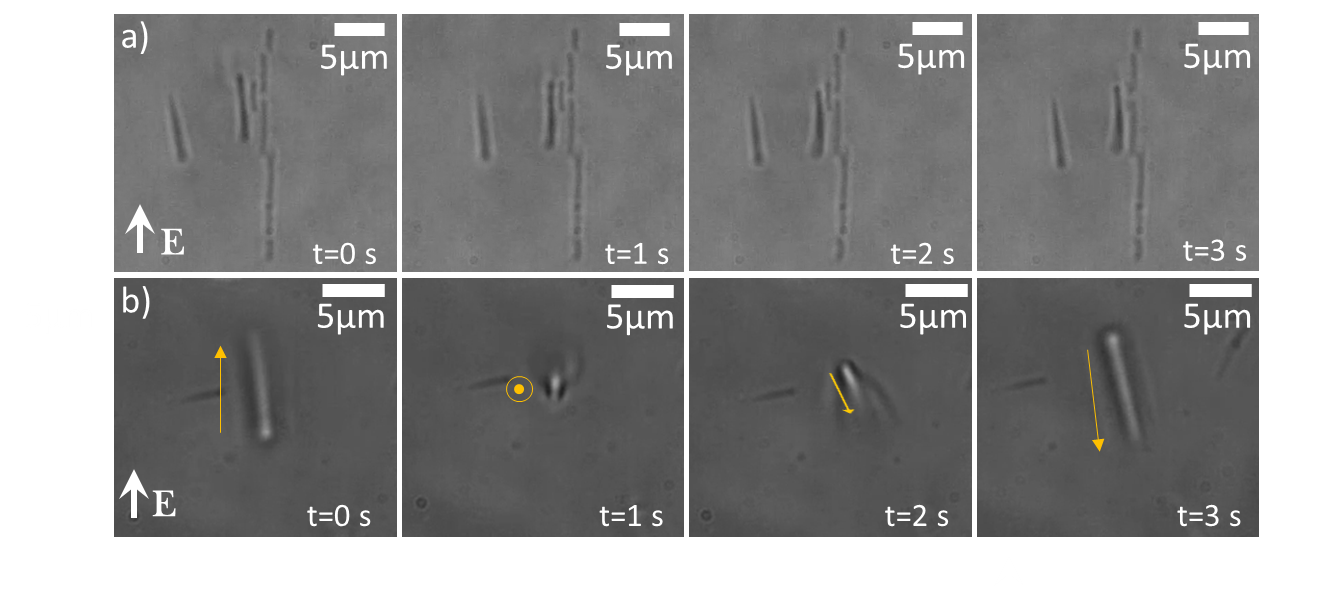


Figure S4: Silica particles in water in EAF 1 MHz 30 V/mm: a) bare particles b) particles with grafted PEI; Coating particles with a grafted polyelectrolyte brush makes them deformable therefore they rotate in EAF.


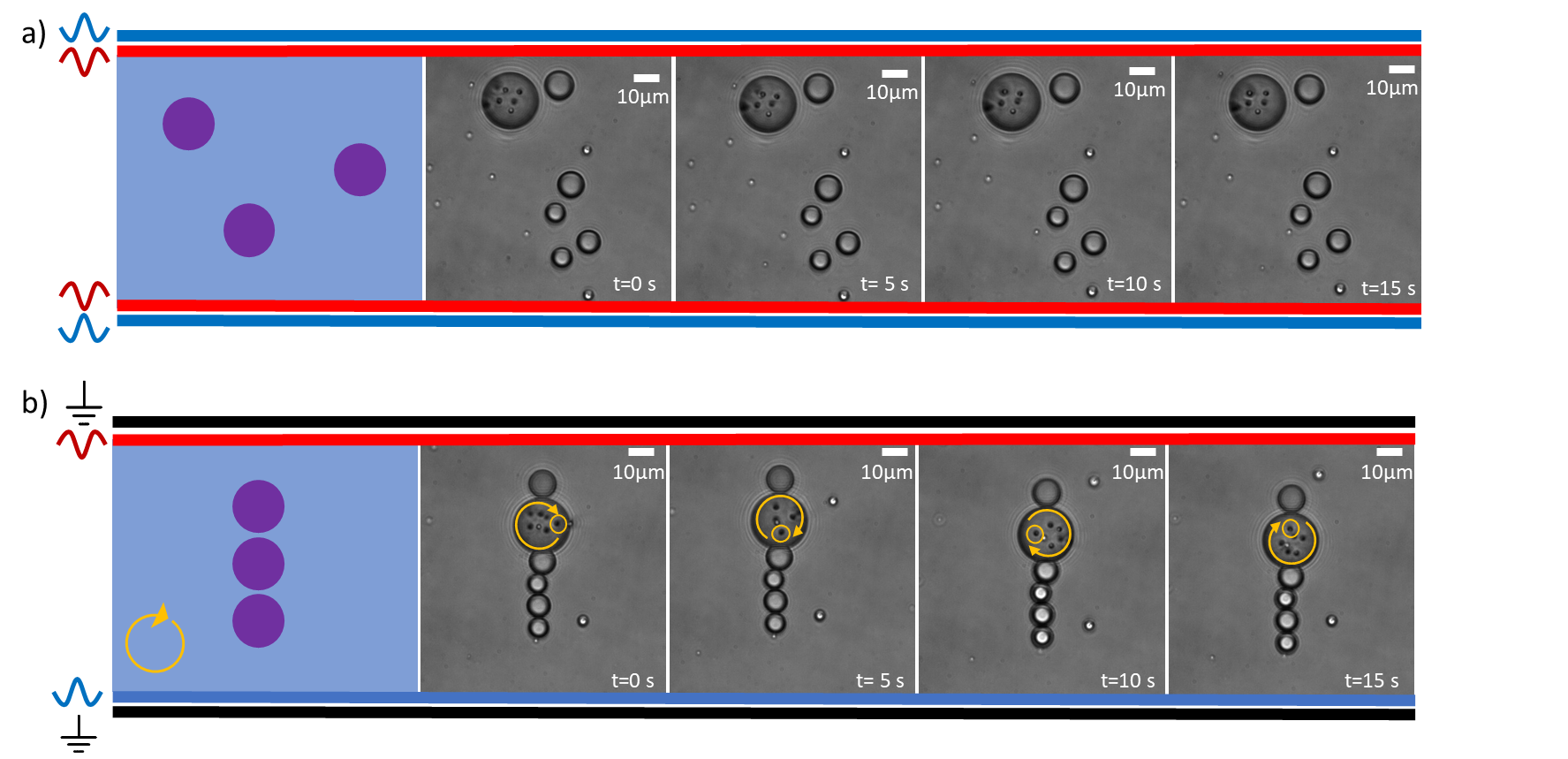

**Silicone oil in water:** Emulsions were prepared by adding 200cSt silicone oil (4130.1 carlRoth) to DI water at room temperature, after which the mixtures were shaken for a minute to separate the oil into droplets. Micron particles were added to the oils in advance to track the movement of these droplets. 1 µm titanium dioxide particles (248576 Sigma-Aldrich) were added to the silicone oil before its addition to water. Bare titanium dioxide particles form aggregates on the oil-water interfaces and detach into the water phase due to their high surface charge. To make the titanium dioxide particles colloidally stable in the oil, we coated them with silica. Using a modified version of the protocol described in [3]: in a typical procedure, 3 g of Tio2 (1 µm) microparticles were dispersed in 120 g of MilliQ water by ultrasonic dispersion (P 30 H) for 15 min, and then added into a solution containing 47.4 g of ethanol, 0.384 g of CTAB (H5882 Sigma-Aldrich), and 1.08 g of 30% ammonia solution. The mixture was stirred mechanically for 30 min at room temperature to prepare a homogeneous solution, and then 1.2 g of TEOS was added drop-wise. The reaction was allowed to proceed for 2 h at 30 °C under constant stirring. The products were collected by centrifugation and washed with MilliQ water and ethanol several times.


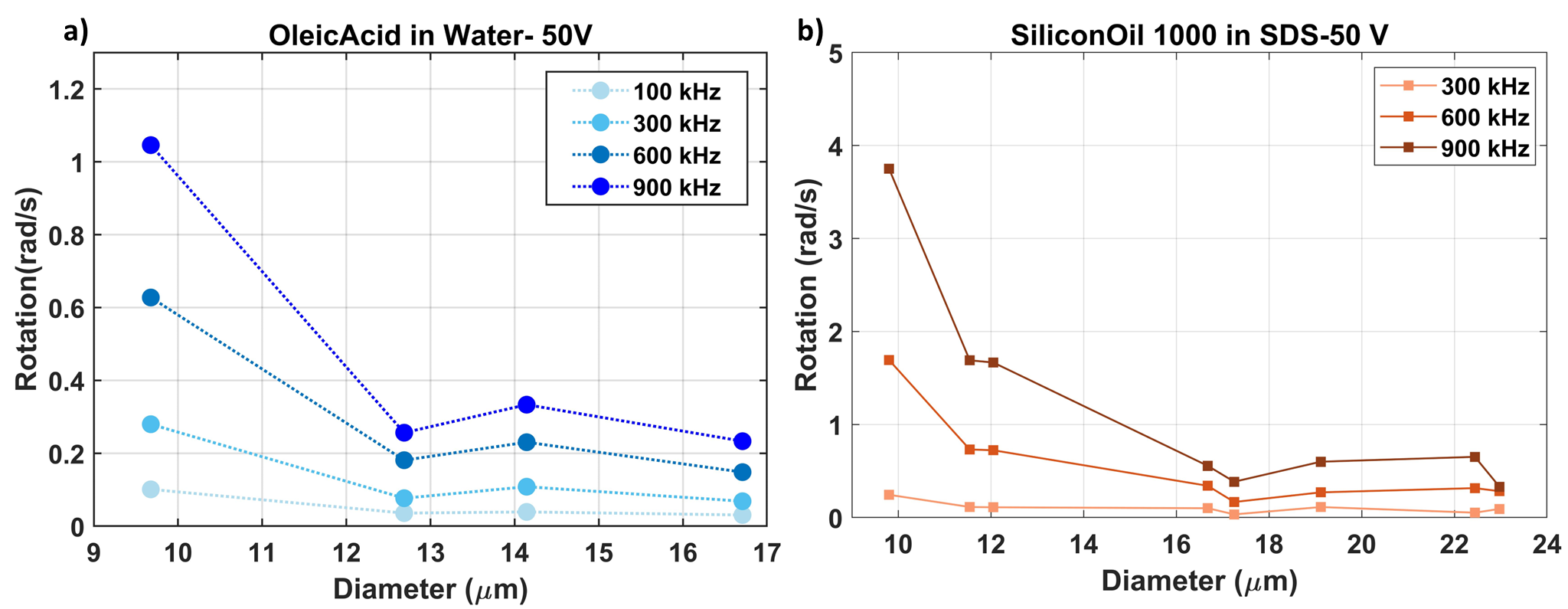


Figure. S6: a) Angular velocity of oleic acid in water per drop at 50 V and different frequencies. b) Angular velocity of silicon oil1000 in 1mM SDS solution and in 3 frequencies of EF per drop diameter. Voltage is fixed at 50V.

**List of movies:**

Movie S1: Rotation and string formation of oleic acid droplets in water in an electroacoustic field. 1 µm polystyrene particles in the droplets are used as tracers to track the rotation.

Movie S2: Strings of oleic acid droplets in water in a silent electric field at different frequencies showing only string formation. 1 µm polystyrene particles in the droplets are used as tracers to track the rotation.

Movie S3: Rotation of silicon oil droplets in water in an electroacoustic field (100-700 kHz, 20 V/mm) and in an acoustic field generated by a piezoelectric actuator (100-700 kHz), 1 µm silica-coated titanium dioxide particles in the droplets are used as tracers to track the rotation.

Movie S4: MCF-7 cell rotation in an electroacoustic field recorded in the middle of the capillary, far from the electrode walls.

1. H. J. Butt and M. Jaschke, *Nanotechnology*, 1995, **6**, 1-7.

2. R. Benítez, V. J. Bolós and J.-L. Toca-Herrera, *The R Journal*, **9**, 291-308.

3. Y. Chen, A. Chen and J. Qin, *RSC advances*, 2017, **7**, 6548-6558.
